# Methylation in cornea and corneal diseases: a systematic review

**DOI:** 10.1038/s41420-024-01935-2

**Published:** 2024-04-08

**Authors:** Yutong Xia, Kuangqi Chen, Qianjie Yang, Zhitong Chen, Le Jin, Liyue Zhang, Xin Yu, Liyin Wang, Chen Xie, Yuan Zhao, Ye Shen, Jianping Tong

**Affiliations:** Department of Ophthalmology, The First Affiliated Hospital of Zhejiang University, Hangzhou, 310003 China

**Keywords:** Biomarkers, Epigenetics

## Abstract

Corneal diseases are among the primary causes of blindness and vision loss worldwide. However, the pathogenesis of corneal diseases remains elusive, and diagnostic and therapeutic tools are limited. Thus, identifying new targets for the diagnosis and treatment of corneal diseases has gained great interest. Methylation, a type of epigenetic modification, modulates various cellular processes at both nucleic acid and protein levels. Growing evidence shows that methylation is a key regulator in the pathogenesis of corneal diseases, including inflammation, fibrosis, and neovascularization, making it an attractive potential therapeutic target. In this review, we discuss the major alterations of methylation and demethylation at the DNA, RNA, and protein levels in corneal diseases and how these dynamics contribute to the pathogenesis of corneal diseases. Also, we provide insights into identifying potential biomarkers of methylation that may improve the diagnosis and treatment of corneal diseases.

## Facts

- Epigenetic modifications have been increasingly linked to the pathogenesis of various ocular diseases, such as keratitis, glaucoma, age-related macular degeneration, and diabetic retinopathy, among others.
- Methylation is a key regulator in the pathogenesis of corneal diseases, including inflammation, fibrosis, and neovascularization, making it an attractive potential therapeutic target.

## Open questions

- What are the major alterations of methylation and demethylation at the DNA, RNA, and protein levels in corneal diseases and how these dynamics contribute to the pathogenesis of corneal diseases?
- Are there any potential biomarkers of methylation that can enhance the diagnosis and treatment of corneal diseases?

## Introduction

As a phenomenon that is beyond genetics, epigenetic changes can dynamically manifest in response to developmental, environmental, and nutritional cues without altering the gene sequence [1, 2], they can influence the regulation of gene expression, phenotypes, and metabolic abnormalities [3]. Currently, three primary mechanisms are recognized to regulate gene expression: DNA methylation (and demethylation), histone modifications and non-coding RNA regulation [4]. With continuous advances in sequencing technology, epigenetics is now being used in various areas of research. Epigenetics offers a partial explanation for diseases [5] including cardiovascular [6], endocrine diseases [7], and autoimmune diseases [8] as well as many other complex pathophysiological processes such as inflammation [9], immunity [8] and neovascularization [10]. Epigenetic factors offer a partial explanation for diseases and may help to explain their onset and progression. Additionally, these factors support emerging epigenetic therapies for diseases [11, 12]. The past decade witnessed the increasing importance of epigenetics in eye development and ocular diseases [13–15]. Unlike inherited genetic modifications, which remain static, epigenetic changes are dynamic and can be influenced by environmental conditions, individual lifestyle, and diseases [16, 17]. Therefore, methylation modifications play a crucial role in the interaction between external factors and the genome.

Methylation is a crucial aspect of epigenetic modifications, whereby a methyl group is transferred from reactive compounds like S-adenosylmethionine (SAM) to other molecules by the action of methyltransferases. Specifically, these enzymes can modify diverse substrates such as DNA [18], RNA [19] and proteins [20]. And these biomolecules can undergo chemical modification through methylation processes, forming methylation products that impact protein functions and regulate gene expression and shutdown [21, 22]. Different forms of methylation can uniquely regulate epigenetic phenomena and play critical roles in cellular behaviors [21]. Clinically, epigenetic modifications have been increasingly linked to the pathogenesis of various ocular diseases, such as keratitis, glaucoma, age-related macular degeneration, and diabetic retinopathy, among others [12, 23, 24]. Furthermore, roles of methylation modifications in ocular physiopathology have also been pointed out [25].

**Fig. 1:**
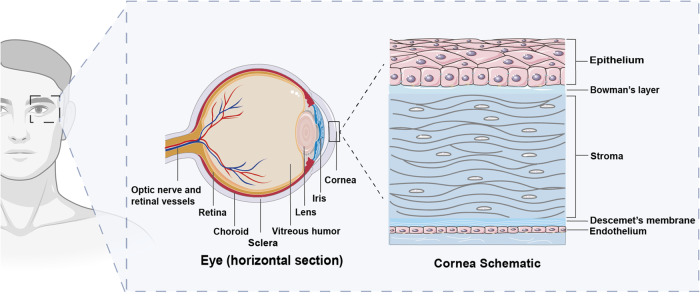
The anatomy of the eye and cornea. The eye is an important visual organ that contains the cornea, lens, vitreous, retina, optic nerve, and other significant structures. The cornea is a transparent tissue at the front of the eye, which is composed of five distinct layers from anterior to posterior: CE, Bowman’s layer, corneal stroma, Descemet’s membrane, and the endothelium. Each layer of the cornea is crucial to its function since it not only acts as the first mechanical and immune barrier to the eye but also transmits and converges external light to the retina to produce vision. (Figure was created with BioRender.com).

The eye is a vital visual organ that comprises several essential structures, including the cornea, lens, vitreous, retina, optic nerve, and others (Fig. 1). The cornea is a transparent tissue located at the front of the eye and consists of five distinct layers, arranged from anterior to posterior: the corneal epithelium (CE), Bowman’s layer, corneal stroma, Descemet’s membrane, and the endothelium (Fig. 1). Functionally, the cornea serves as the ocular initial mechanical and immune barrier, transmitting external light to the retina to generate vision. Moreover, it not only transmits essential light for vision but also refracts light, providing focus to images [26]. Therefore, any damage to or illness of the cornea can result in severe vision loss or blindness [27]. Currently, although methylation has been extensively studied in posterior segment diseases, its potential role in anterior segment diseases has not been much emphasized. Moreover, recent researches have highlighted the impact of DNA methylation, N6-methyladenosine (m^6^A), and other methylation mechanisms on corneal functions, including cell migration, as well as corneal-related diseases, such as keratitis [28, 29], corneal would [14, 30, 31]. Despite the relative wealth of information on the transcriptional regulation of corneal cells and differentiation, reviews describing the potential of methylation in corneal diseases (CDs) are scarce and it is unclear how methylation modifications exactly affect corneal gene expression and CDs.

Our review aims to summarize the role of methylation in CDs, emphasizing the importance of DNA, RNA, and histone methylation in the cornea. Additionally, we provide insight into the pathogenesis of methylation-related CDs to offer new perspectives for their treatment.

## Methylation mechanism and function

### Classifications of methylation

The methylation pattern is determined by a dynamic balance of methylation and demethylation [32, 33], regulated by specific enzymes: “writers” or methyltransferases, “erasers” or demethylases, and recognized by “readers” or methylation-dependent binding proteins [21]. Various forms of methylation modifications, along with related modification factors, can play critical roles in corneal functions and health (Table 1).

**Table 1:** Overview of various methylation studies in corneal diseases.

Core event	Methylation type	Tissue type	Conclusion/Significance	Contributors
KTCN	DNA methylation	Human	It revealed unique DNA methylation patterns in human KTCN corneas.	Kabza [120]
Corneal wound	DNA methylation	Mice hCECs	1. DNMT1 and DNMT3B expression was significantly upregulated during CEWH. 2. miR-200a and CDKN2B were identified as molecular targets of DNA methylation and as having a causal connection with DNMT1.	Luo [62]
		Mice	The involvement of DNMT3B-madiated DNA methylation and PI3K/AKT/mTOR signaling modulation in Alkali burn.	Li [31]
	RNA methylation	Mice HUVEC	FTO regulates ocular angiogenesis in an m^6^A-YTHDF2-dependent manner.	Shan [30]
		Mice HUVEC	METTL3 enhances the translation of specific target genes (*lrp6*, disheveled 1) which is mediated by YTHDF1.	Yao [134]
		Mice hCECs	NSUN2-mediated 5-Methylcytidine modification of UHRF1 mRNA modulates CEWH	Luo [176]
	Histone methylation	Human	Maspin synthesis is downregulated in corneal stromal cells by methylation of maspin promoters and histones.	Horswill [106]
		Mice HUVEC	Inhibition of *EZH2* alleviated corneal angiogenesis by inhibiting FoxO3a/PI3K/AKT/mTOR signaling pathway.	Wan [182]
		Mice hCECs	Knockdown of *SUV39H1* regulated the p27 expression level and reduced H3K9me3 marks at p27 promoter.	Yang [175]
		Mice CFs	EZH2 is a new target to inhibit corneal scarring by activating anti-fibrosis genes.	Liao [110]
Keratitis	RNA methylation	Mice	It represents the pioneering exploration of m^6^A modification profiles in experimental fungal keratitis.	Hu [28]
	Histone methylation	Mice hCECs	Inhibition of *Dot1L* prevented corneal oxidative stress and inflammation through the p38 MAPK pathway in HSK.	Wan [107]
Corneal dystrophy	DNA methylation	Human	1. DNA methylation patterns play role in loss of corneal transparency. 2. Impaired fluid transport, cellular homeostasis, and cytoskeletal organization associated with gene methylation levels.	Khuc [186]
		Human	Snail and ZEB1 are upregulated by miR-199B hypermethylation	Pan [187]
	Histone methylation	Human	Knockdown of both *MLL1* and *SET7/9* significantly blocked the TGFβ1-induced gene expression and inhibited TGFβ1-induced changes in promoter H3K4me1/3 levels.	Maeng [185]
Brittle cornea syndrome	Histone methylation	Human	H3K9me2 at these PRDM5-target genes in fibroblasts and demonstrate that the BCS2 mutation p.Arg83Cys diminishes interaction of PRDM5 with repressive complexes.	Poter [205]
Diabetic keratopathy	DNA methylation	Human	1. Significant differential DNA methylation between diabetic and non-diabetic limbal epithelial cells. 2. Wnt-5a promoter was hypermethylated in diabetic limbal epithelial cells accompanied with markedly decreased Wnt-5a protein.	Shah [14]

#### DNA methylation

Actually, DNA methylation has been the most extensively studied epigenetic phenomenon to date and has been significantly translated into clinical applications for early diagnosis and therapy [21, 34]. Mechanistically, as a modification that does not alter the DNA sequence, DNA methylation plays a critical role in tissue-specific gene expression, genomic imprinting, chromosome stability, and more [35]. DNA methyltransferases (DNMTs) are responsible for catalyzing DNA methylation, and act as the transfer of methyl groups from SAM to the fifth carbon of DNA cytosine residues [36]. This results in a chemical modification where methyl groups are covalently bonded to the cytosine residues [37]. Different types of DNA methylation include 5-methylcytosine (5mC), 5-hydroxymethylcytosine (5hmC), N6-methyladenine and others [38, 39], with 5mC being the most common type, and its DNA methylation status is influenced by specific regulatory molecules.

**Fig. 2:**
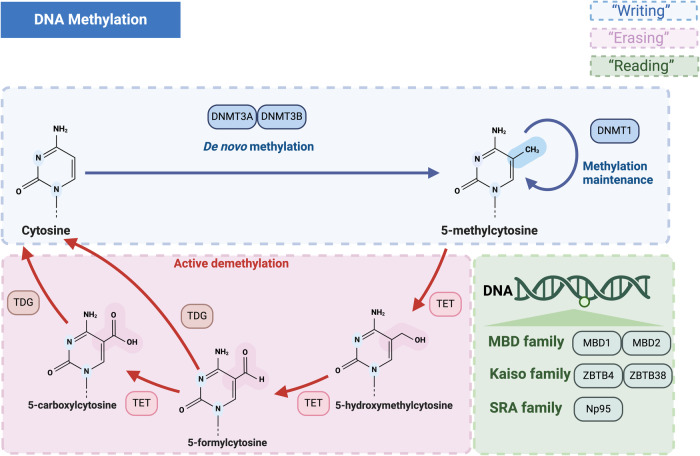
The primary type and mechanism of DNA methylation. DNA methylation is catalyzed by DNMTs, facilitating the transfer of methyl groups from SAM to the fifth carbon of DNA cytosine residues. This results in the chemical modification of methyl groups through covalent bonding. The role of methylation modifiers such as “writers” (DNMT1, DNMT3A, DNMT3B), “erasers” (TETs) and “readers” (MBD1-2), have been widely reported, with some of them also participating in CDs. (Figure was created with BioRender.com).

The “writer” proteins are key players in DNA methylation and include DNMT1, DNMT3A, DNMT3B and DNMT3L (Fig. 2). DNMT1 is critical for maintaining normal levels of DNA methylation. It targets double-strands DNA molecules that have only one methylated strand, playing a role in methylating the newly synthetic strand during semiconservative DNA replication [33]. DNMT3A and DNMT3B are two de novo methyltransferases that are capable of catalyzing the initial methylation of CpG sites [40]. The DNMT3L enzyme promotes DNMT3A/B but is itself catalytically inactive [35, 37, 41]. Previously, DNA demethylation was considered a passive process. However, the discovery of the Ten-eleven translocation dioxygenases (TETs) protein family reveals an active process that plays an important regulatory role (Fig. 2) [42, 43]. Regarding “reader” proteins, the three primary types of DNA methylation-binding proteins are members of the Methyl-CpG-Binding Domain (MBD), Kaiso, and Set and Ring Finger-associated (SRA) families [44–48] (Fig. 2).

Functionally, DNA methylation is crucial for preserving the stability of eukaryotic genomes and controlling vital physiological processes, such as the cell cycle and cellular development [33, 49, 50]. Numerous factors regulate gene expression, and the role of methylation in gene expression has been disputed. DNA methylation can regulate gene expression in many ways such as altering DNA conformation, enhancing DNA stability, and modifying chromatin structure. Specifically, promoter methylation can alter gene expression, leading to various pathogenic processes that ultimately result in numerous eye diseases, including CDs [31, 51].

#### DNA hypomethylation and hypermethylation

DNA hypomethylation is a significant DNA methylation state, alongside hypermethylation. It generally describes a relative situation where there is a decrease from the “normal” level of methylation [52]. Loss of methylation leads to euchromatin formation associated with highly transcriptionally active regions of DNA, increasing the risk of genomic instability [53]. In contrast to DNA hypomethylation, the addition of methyl groups to an excessive number of cytosine bases at gene promoters leads to repressed transcriptional activity [54]. Hypomethylation and hypermethylation play crucial roles in regulating gene expression, maintaining genome stability, and controlling important biological processes [55–57].

#### The role of CpG islands (CpGi) in DNA methylation

Methylated cytosines are non-randomly distributed in the genome. In mammals, DNA methylation primarily occurs at the cytosine 5’ of the CpG dinucleotide [58]. CpG dinucleotides have been demonstrated to be scattered throughout the DNA of mammals or in clusters known as CpGi [59]. Over 70% of genes, including housekeeping genes, have their promoters located within CpGi [60, 61]. Methylation reduces the expression of downstream target genes [62], but has the opposite effect in specific cases [63]. Methylation levels at CpG sites can vary under different conditions. The density of CpG dinucleotides, the nature of the target gene, its location and degree of methylation all affect the outcome of methylation [64–66]. Methylation of CpGi in or around gene promoters is an essential pathway to gene silencing and imprinting [67–69]. In general, gene promoters of active genes have demethylated CpG regions, whereas silenced or low-expressed genes have hypermethylated regions. By specifically methylating certain CpG sites, certain circumstances may cause some genes to be transcriptionally suppressed [31]. DNA methylation was once thought to indicate transcription repression, and specific transcription factors (TF) are needed for gene transcription in eukaryotes. Methylation of CpGi can prevent TF binding and silence genes [40]. Conversely, TF binding can also prevent DNA methylation [70]. There is ongoing debate about how epigenetic modifications are inherited in mammals. A recent study revealed that methylation of CpGi can be passed down across generations by changing DNA methylation in mice. This discovery leads to new research on the role of methylation [71].

### RNA methylation

**Fig. 3:**
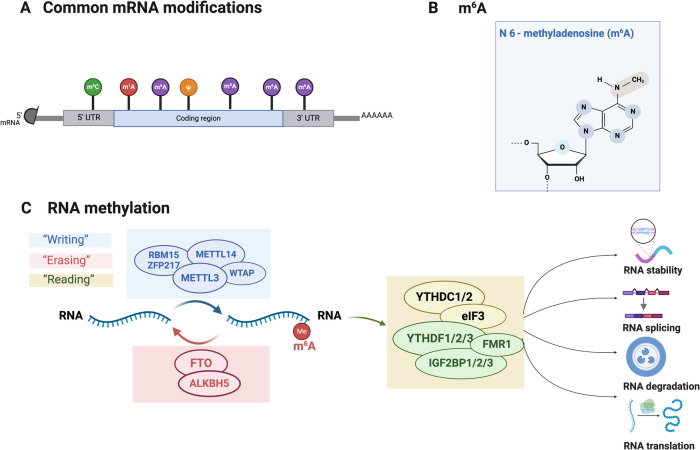
The primary types and mechanisms of RNA methylation. **A** The methylation modifications occurring at different sites of mRNA reflect the diversity of RNA methylation. **B** The most representative RNA methylations are m^6^A. **C** m^6^A is catalyzed by the “writers” (METTL3/METTL14/WTAP) and removed by FTO and ALKBH5 “erasers”. In addition, YTHDC1, YTHDC2 and YTHDF1/2/3 serve as m^6^A methylation-dependent binding proteins, acting as “reader” to regulate RNA stability, splicing, degradation, and translation functionalities. (Figure was created with BioRender.com).

As a crucial regulator of transcriptional expression, RNA methylation occurs in many types of RNAs, including messenger RNA (mRNA) (Fig. 3A) and transfer RNA. Functionally, RNA methylation and its associated signaling pathways are involved in numerous biological activities, such as cell differentiation, the stress response [72]. RNA methylation occurs in at least 150 forms across diverse RNA molecules [73]. Among them, m^6^A and C5-methlcytidine are the most widely studied. m^6^A refers to the methylation modification of the nitrogen atom (N) at position 6 of adenine (Fig. 3B), and it represents the most prevalent mRNA modification in humans and other mammals [74] (Fig. 3A), accounting for up to 50% of RNA methylation [75]. There is specific research on m^6^A in the eye, making it a popular area of research in biology [76].

“Writer” proteins influence reversible RNA methylation similarly to how they regulate DNA methylation. Methyltransferase-like 3 (METTL3), Methyltransferase-like 14 (METTL14), and Wilms tumor 1 associated protein (WTAP) [77] (Fig. 3C) make up the majority of the m^6^A methyltransferase complex. Their primary function is to catalyze the m^6^A modification of adenosine on mRNA [78]. METTL3, as the pioneer RNA methyltransferase discovered, plays essential catalytic roles in m^6^A methylation, and METTL14 may augment METTL3’s catalytic activity [79].

The discovery of Fat mass and obesity-associated protein (FTO) as a m^6^A demethylase sheds light on the fact that m^6^A can be dynamically regulated, suggesting its importance in normal development and the pathogenesis of diseases [80]. Since then, FTO and AlkB homolog 5 (ALKBH5), both belonging to the AlkB family of Fe(II)/a-ketoglutarate-dependent dioxygenases, have been recognized as “eraser” proteins that remove m^6^A RNA modifications [81, 82] (Fig. 3C). Demethylases remove methylation from m^6^A-modified bases, regulating intracellular homeostasis and cellular damage repair [21, 81]. This highlights the dynamic and reversible nature of m^6^A modification. Specific biological functions can be carried out through this process, m^6^A-modified mRNAs necessitate particular RNA-binding proteins referred to “reader” proteins which include IGF2 mRNA binding proteins (IGF2BP1/2/3), the YTH domain protein family (YTHDC1/2, YTHDF1/2/3), eukaryotic initiation factor 3 (eIF3) and others [83] (Fig. 3C). The YTH domain, discovered within over 200 proteins, including YTHDC1, YTHDC2, YTHDF1, YTHDF2, and YTHDF3 [84], binds proteins and recognizes m^6^A-modified bases, initiating pathways for RNA degradation and miRNA processing. This is a common type of RNA base modification that mainly regulates RNA stability, splicing, degradation, translation, and other processes [85, 86] (Fig. 3C).

### Histones methylation

Post-translational modifications (PTMs) are a frequent means of modifying proteins and changing their functions. Methylation, as a type of protein PTMs, significantly impacts cellular physiology and pathogenesis [87]. It affects the structure and activity of the modified protein as well as the interaction with other proteins [88], thereby regulating the translation, localization and signal transduction of the protein.

**Fig. 4:**
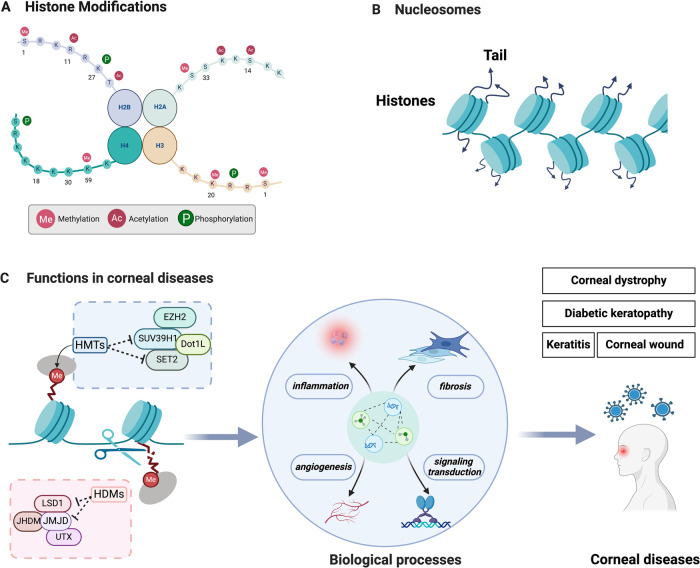
The mechanisms of histone methylation. **A** Each of the histones H2A, H2B, H3, and H4 has two molecules in the histone core particle. Histones are highly conserved proteins with flexible N- and C-terminal domains as well as a conserved globular domain. **B** Histones combine with DNA to form nucleosomes, which then assemble into chromatin in eukaryotic cells. Most histones have globular cores, with flexible “tails” extending from their N-termini. **C** Histone methylation is regulated by HMTs such as SUV39H1, DOT1L, EZH2 and HDMs such as LSD1 and JMJD. Several studies have explored the association between the biological processes during CDs and corresponding HMTs and HDMs. (Figure was created with BioRender.com).

Indeed, methylation can occur on both histones and non-histones [87, 89]. Histones bind with DNA to create nucleosomes, which consist of 147 base pairs of DNA coiled around the histone core particles. Sequences of nucleosomes make up chromatin in eukaryotic cells. The histone core particle is composed of two molecules each of the histones H2A, H2B, H3, and H4. Histones are proteins that are highly conserved, having flexible N- and C-terminal domains along with a conserved globular domain (Fig. 4A). Most histone cores are globular, with less rigid N-terminus tails that can be modified by various types of modifications such as acetylation, methylation, and phosphorylation [90–93] (Fig. 4B).

The histone modifications are found in various residues of histone H3 and histone H4 [87]. It is regulated by histone methyltransferases (HMTs) [94] and histone demethylases (HDMs) (Fig. 4C). To be more specific, lysine methyltransferases (KMTs) are responsible for histone methylation, which trigger monomethylation, dimethylation, and trimethylation [95]. The majority KMTs contain the SET domain, which forms the methylation complex with the help of certain structural subunits and sustains the activity of KMTs [96]. HDMs can be roughly divided into two groups: Lysine-specific demethylase (LSD) and JmjC domain-containing family (JMJD) (Fig. 4C). Specifically, LSD1 can remove the mono-dimethylation modification of histones H3K4 and H3K9, while JmiC can remove the trimethylation modification of lysine [97, 98]. The discovery of the first histone demethylation modifying enzyme, LSD1, occurred in 2004 [99]. Henceforth, the dynamic regulation of histone methylation via histone methylases and demethylases was brought into the limelight. Methylated histones are recognized by proteins with methyl-binding domains [21, 100]. Functionally, as the most common protein modification, it can affect cell fate in ways other than just at the transcriptome or protein level [101]. Histone methylation can potentially repress or even activate transcription, depending on lysine that gets methylated. To regulate gene transcription, methylation of histones H3 and H4 occurs at distinct sites and varying degrees. The scientific community has widely accepted that the activation of genes is attributed to trimethylation or dimethylation of H3K4, H3K36, and H3K79, while methylation of histone H3K9 is known for its association with gene repression [102]. Additionally, protein methylation modifies arginine and lysine residues in non-histone proteins to regulate cellular signal transduction via MAPK, WNT, BMP, JAK-STAT, p53 and NFkB pathways [87, 103, 104]. These discoveries have furthered our understanding of epigenetic regulation. However, it is poorly understood how HMTs and HDMs act in the mammalian eye, multiple studies have investigated the connection between transcription and expression of specific genes during CDs, along with corresponding HMTs and HDMs involved [105–107] (Fig. 4C). For instance, the transcriptional activation of Enhancer of Zeste Homolog 2 (EZH2), which is a fundamental element of HMTs, triggers histone H3 lysine 27 trimethylation [108, 109] which is recognized to have a significant impact on corneal scarring [110]. Similarly, altered expression levels of Disruptor of telomeric silencing-1 like (Dot1L) which is correlated with H3K79 that results in keratitis [107]. We strongly believe that further investigation into protein methylation modification should be undertaken in CDs.

Different methylation levels correlate with disease severity, and they can act as vital epigenomic markers for the development of effective diagnostic, prognostic and predictive biomarkers for diseases such as cancers [111], neurological diseases [112]. Recent studies have focused more on the effects of methylation modifications in ocular diseases [113]. Our review offers an in-depth exploration of the topic by covering a broad range of processes, including CE repair, ocular fibrosis, and the oxidative stress and inflammatory response caused by different methylation levels. we specifically highlight the significance of methylation modification in relation to the cornea.

## Methylation in corneal physiology and pathology

### Modification factors vary in cornea

**Fig. 5:**
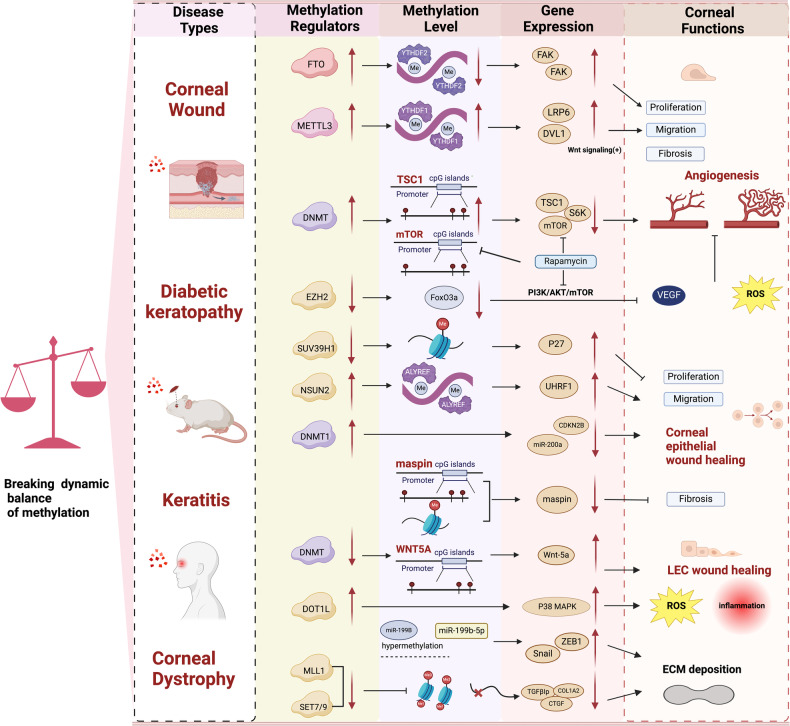
The methylation balance is disrupted in corneal diseases. Maintaining the equilibrium between methylation and demethylation is therefore necessary for sustaining proper cornea function. In corneal diseases, methylation modifications can trigger changes in corneal function and related pathological processes. Regulatory factors for methylation modification can cause changes in methylation levels which, in turn, can affect gene expression. Ultimately, these changes can lead to neovascularization, activation of ROS, and increased expression of inflammatory-related factors. (Figure was created with BioRender.com).

The role of methylation modifiers such as writers (DNMT1, DNMT3A, METTL1, METTL3), erasers (TET1, FTO and ALKBH5) and readers (Methylated CpG binding protein 2, YTHDF1-3, YTHDC1-2) in ocular tissues have been widely reported [81, 114–117]. The expression of these methylation modifiers, namely DNMT1, DNMT2, DNMT3A, DNMT3L, and FTO, can vary during both normal corneal physiological activities and pathological processes [28, 30, 118] (Tab.1). Specifically, DNMTs catalyze DNA methylation modifications in the eye and are involved in various tissues of the preocular segment, including the human cornea, conjunctiva, lens anterior capsule, trabeculae, and related cells. In particular, DNMT1, 2, 3A, and 3L proteins are expressed in the human cornea, with DNMT2 preferentially present in corneal endothelial orientation [118]. Therefore, studying the function of DNMTs in the cornea is crucial. Moreover, different methylation regulators may also be expressed differently in different CDs [119]. Thus, it appears that factors that modify methylation can impact corneal function under both healthy and pathological circumstances. Additionally, these factors can also regulate corneal cell function by activating and expressing specific genes associated with methylation modifications (Fig. 5).

### Regulation of corneal physiology and pathogenic processes

It is crucial to understand how methylation modification affects ocular cellular physiology, and pathology. The majority of ocular diseases in humans have also been linked to methylation dysfunction [120] and CDs continue to be a major cause of vision loss worldwide. The cornea, a transparent tissue with refractive capacity, has to maintain the homeostasis of five layers in order to function normally [121]. These layers are the epithelium, Descemet’s membrane, corneal stroma, Bowman’s layer, and a monolayer of metabolically active but mitotically inactive endothelial cells [27, 122]. Every layer of cells is vital for the proper functioning of the cornea, the epithelium serves as a protective barrier and helps to maintain a smooth surface and Bowman’s layer provides additional support to the stroma below it. The corneal stroma is the thickest layer and is responsible for the corneal strength and transparency. Descemet’s membrane acts as a basement membrane and provides support for the endothelial cells, which regulate fluid balance in the cornea [27, 123, 124]. Without any one of these layers, the corneal normal functioning would be compromised.

Methylation regulators may affect the mechanisms of corneal physiology, including corneal differentiation, pathogenic processes, and homeostasis. According to Sasamoto et al., knockdown of *TET2* in CECs led to a considerable reduction in 5hmC peak distribution, and effected proteins involved in corneal differentiation, including KRT78, MYEOV and MAL [125]. Furthermore, recent research has shown that methylation-induced genetic differential status is linked to the differentiation of induced pluripotent stem cells into CECs [126]. Induced pluripotent stem cells produced from corneal limbal epithelium cells are more likely to differentiate into limbal-like stem cells than those derived from fibroblasts, possibly due to epigenetic methylation changes in genes related to limbal epithelium cells (such as *NTRK1*, which codes for TrkA) [127]. Furthermore, it appears that DNA methylation regulatory factors are closely related to corneal functions such as enhancing the expression of tight junction-related genes like claudin 6 and claudin 9 [128].

Methylation modifications play crucial roles in the pathophysiological processes of several complex CDs (Table 1 and Fig. 5), including inflammation [129], immunity [130], neovascularization [30] and stromal remodeling [131] (Fig. 5). For instance, Luo et al. discovered that upregulation of DNMT1 and DNMT3B during corneal epithelial wound healing (CEWH) affected corneal cell proliferation and migration [62]. Regarding interferon’s function in innate immunity, the cornea is not an exception [130]. In addition, excessive METTL3 promotes m^6^A methylation to decrease interferon synthesis, implying that methylation negatively modulates interferon response [132].

Abnormal angiogenesis is a prominent feature of many CDs, including corneal dysgenesis. The central corneas are normally avascular, but they become vascularized when they are exposed to inflammation, infection, or hypoxia. Under certain pathological conditions, the level of FTO through m^6^A mRNA demethylation is increased in neovascularized corneas, specifically, silencing *FTO* increases m^6^A methylation levels in proangiogenic genes, such as *FAK*. This results in reduced RNA stability and faster RNA degradation via YTHDF2, which attenuates suture-induced neovascularization [30, 133]. In another study, the knockout of *METTL3* inhibited corneal neovascularization in vivo, *METTL3* enhances the translation of specific target genes, including *lrp6* and disheveled 1. This enhancement is mediated by YTHDF1. Moreover, the involvement of these genes suggests that they may play a role in regulating WNT signaling [134]. Additionally, knocking out *METTL3* in corneal limbal stem cells promotes the proliferation and migration of in vivo cells, resulting in fast repair of corneal injury [135, 136]. During corneal injury, CECs also migrate more rapidly. Corneal limbal stem cells possess the ability to regularly renew and differentiate, and they migrate to the central cornea to replace damaged or dead CECs [137]. This research highlight the crucial role of m^6^A in regulating corneal injury repair and offers new insights for the treatment of CDs [136].

Furthermore, infection, trauma, chemical or surgical damage to the cornea can cause fibrosis in the cornea, leading to impaired vision. Some studies discovered that corneal fibrosis may be related to methylation levels [138, 139]. Promoter and histone methylation could regulate the differentiation of keratinocytes into wound-healing fibroblasts. Maspin enzyme, which is largely epithelial in origin but is also present in corneal stromal keratinocytes, is hypothesized to be downregulated during the transformation of keratocytes to fibroblasts. This involved promoter methylation on a CpGi and histone methylation of the *maspin* gene [140, 141]. The scarring of the cornea is caused by fibrosis of the tissue at the end of the process of corneal injury, which may result in vision loss [121]. However, the mechanism behind corneal scarring is still not well understood, and there is no specific treatment to alleviate or cure corneal scarring. Current treatment modalities are mainly corneal transplantation [142]. Recent efforts have focused on understanding the role of histone methylation in corneal scar formation. EZH2 has been shown to be upregulated in certain fibrotic diseases tissues [143]. A study conducted by Liao et al. revealed that the expression of EZH2 was upregulated in vitro (cellular models of corneal myofibroblasts), high-throughput transcriptome sequencing revealed that blocking EZH2 may inhibit corneal fibroblasts (CFs) activation by inducing the expression of antifibrotic genes [110]. This process suggests that effects of gene promoter and histone methylation may be associated with corneal fibrosis.

### Methylation and demethylation in dynamic equilibrium

Methylation and demethylation processes are in a state of dynamic equilibrium. The methylation process can be influenced by various factors, including genetics, individual characteristics, and environmental influences such as aflatoxin B1 and air pollution. While genetic factors are known to affect susceptibility, non-genetic risk factors like DNA methylation modifications, histone modifications, and inflammatory risk factors are also gaining attention in this regard [144–147]. Methylation levels in tissues are variable and dynamic [148, 149], with a balance between establishing and eliminating methylation [33], which is mediated by methyltransferases and demethylases [150] (Fig. 5).

#### DNA methylation and demethylation

During DNA demethylation processes, the commonly occurring demethylase TET protein tightly regulates DNA methylation modifications by promoting active DNA demethylation and dynamically regulating the levels of 5mC and 5hmC [151], thereby regulating the activation of specific gene expression [152]. DNA methylation can turn off gene expression, and demethylation can turn on gene expression [153]. Indeed, specific TF binding sites are present after some promoter demethylation. These sites bind to non-coding DNA sequences surrounding or covering the promoter region, affecting RNA polymerase function, and blocking gene activation. In the case of mTOR gene promoter methylation caused by alkali burns (Fig. 5), rapamycin may erase or diminish the methylation so that certain TF binding sites become accessible to the transcription factor [31].

#### RNA/Histone methylation and demethylation

The dynamic character of methylation is implied by the dynamic changes in methylation regulatory factors such UHRF1 and TET3 [66, 154]. Regulatory molecules that maintain the dynamic balance of methylation (such as METTL3/FTO) are expressed in different tissues of the eye as well as in cornea [155]. Maintaining the equilibrium between methylation and demethylation is critical for proper cornea function. For instance, the methyltransferase METTL3 plays a biological role in maintaining homeostasis in mouse T cells and in differentiated T cell-mediated pathogenesis [156]. Disrupting this balance may contribute to the development of corneal immune diseases. The different sites and patterns of histone methylation can evolve many methylation modification patterns in cornea, which increase the complexity and diversity of gene expression (Fig. 5). It is the duty of HMTs and HDMs to maintain the level of histone methylation.

Furthermore, methylation is dynamic in different stages of the host. For instance, post-translational methylation modifications in aging ocular tissue are dysregulated. Several methylation metabolites can accumulate in aged corneas, and other ocular tissue [157]. The increased accumulation of methylated metabolites can potentially impact methylome metabolism.

Overall, understanding the dynamic nature of methylation and demethylation processes along with their regulation by factors like genetics, environment, and enzymatic activities is crucial for comprehending the complex mechanisms involved in gene expression and tissue function.

## Methylation in CDs

### Keratitis

Current studies on methylation in keratitis focus mainly on Herpes simplex keratitis (HSK), which is caused by the highly prevalent Herpes simplex virus (HSV) [158]. Ocular disease brought on by HSV-1 infection is typically manifested as HSK [159]. In developed countries, HSV infection is a leading cause of corneal blindness [160]. Various pathological processes, such as inflammation, oxidative stress, neovascularization, and endothelial damage, can result in corneal damage, clouding, vision loss, and even blindness [159, 161]. Unfortunately, there are currently limited treatment options available. Recent studies have demonstrated that methylation-related mechanisms tightly regulate the establishment of latency and reactivation of HSV-1. Due to the continuous expression of latency-associated transcripts and simultaneous transcriptional suppression of lytic genes, histone methylation modifications are involved in the differentiation of active and inactive genomic regions. This suggests the significance of histone methylation in HSV-1 [105, 162]. As more is understood about the role that HKM plays in herpes virus, it could become an increasingly important epigenetic target for treating ocular HSV infections.

When the host is infected with the virus, the innate and adaptive immunity systems of the host inhibit HSV-1 replication and act as anti-infective role. However, the virus also damages the host’s immune system, leading to herpes-related immune inflammatory response in the corneal stroma that is mainly regulated by the pro-inflammatory CD4 Th1 and Th17 cells [163, 164]. A crucial balance exists between inflammatory T cells and regulatory T cells (Tregs), such as Foxp3^+^ CD4 T cells between other pro-inflammatory CD4 T cell subsets [163, 165, 166]. Studies have demonstrated that the specific demethylation region in the highly conserved intron 2 of Tregs is vital for the transcription of the *Foxp3* gene. When this region is demethylated, transcription factors Ets-1 and CREB can bind to each other and act as enhancers of sequential transcription of the *Foxp3* gene [167, 168] and *Foxp3* gene expression is controlled by CREB/ATF sequence-specific binding and CpGi DNA methylation, etc. [168]. Moreover, 5-Azacytidine (5-Aza) covalently binds to cysteine residues at DNMT catalytic sites, ensuring the normal function of Tregs to reduce corneal inflammation. An interesting finding of this research was the 5-Aza therapy enhanced the function and suppressive activity of Tregs, reducing lesions and effectively controlling virus-induced inflammation [29]. Furthermore, oxidative stress is considered as one of the critical factors in the pathogenesis of CDs [169], including keratitis. In the progression of HSK, the activity of superoxide dismutase continues to decrease, while the levels of malondialdehyde and hydrogen peroxide continue to increase [170]. Several investigations have indicated that Dot1L may regulate oxidative stress, because Dot1L plays a specific role in catalyzing the methylation of H3K79 in the targeted genes [171, 172]. Moreover, Dot1L can be used to regulate the expression of pro-inflammatory factors that involved in the p38-MAPK pathway such as IL-1β, matrix metalloproteinases (MMP)-1, MMP-2, IL-6 and MMP-9 in HSK [107]. Therefore, reducing the formation of reactive oxygen species (ROS) decreased corneal oxidative stress and inflammatory response, suggesting that Dot1L could be a potential target for therapeutic interventions to treat HSK.

Furthermore, in a study of global m^6^A levels, Hu et al. discovered that the expression of METTL3 was considerably higher in fungal-infected corneas than in normal corneas. This suggests that m^6^A methylation may be implicated in the transduction pathways of fungal keratitis by regulating several critical signals, including the PI3K-Akt signaling pathway [28]. Above all, inhibiting methylation modification may limit pro-inflammatory and pro-oxidative stress in numerous ways, thereby preventing the onset of keratitis.

### Corneal injury

The CE, which is the outermost layer of the cornea, can regenerate and is especially sensitive to physical, chemical, and pathological stimuli [173, 174]. CEWH is crucial for repairing the damage caused to the cornea and restoring its integrity and transparency.

Recent studies have demonstrated that DNA methylation, RNA methylation and histone methylation modifications can regulate CEWH [62, 175]. Luo et al. found that corneal epithelial injury led to higher levels of DNMT1 and DNMT3B expression, as well as significant overall DNA hypermethylation modifications. Upregulation of DNMT1 significantly increased the rate at which CE injuries healed, as well as the ability of hCECs to proliferate and migrate. The proposed mechanism is that DNA hypermethylation decreases miR-200a and Cyclin-dependent kinase inhibitor 2B (CDKN2B) expressions [62]. Furthermore, modifying mRNA with 5-Methylcytidine via *NSUN-2* also affects CEWH. Knocking down *NSUN-2* delayed CEWH and inhibited hCECs proliferation and migration in vitro [176].

DNA and RNA methylation, along with the upregulation of histone methylation transferase suppressor of variegation 3–9 homolog 1 (SUV39H1), are involved in CEWH following corneal injury. Specifically, SUV39H1 is crucial for controlling CECs proliferation by suppressing p27 through H3K9me3-mediated mechanisms during CEWH. Epigenetic modifications like SUV39H1 hold promise as potential therapeutic interventions to speed up the process of corneal repair [175].

Corneal chemical injury is a common ophthalmic emergency, and alkalis can penetrate ocular structures with high capacity, resulting in burns that have a significant impact on vision. Clinically, corneal alkali burns (CAB) can lead to a range of complications such as delayed epithelial healing, conjunctival scar formation, dry eye disease, vascularization and corneal clouding [177]. The major pathological features following CAB are corneal vascularization, inflammation, and fibrosis [178, 179]. Several studies have shown that rapamycin, a mTOR receptor inhibitor, can reduce corneal turbidity and neovascularization in CAB by various signaling pathways like TGF-1/ERK [180, 181]. An interesting study by Li et al. discovered that DNMT3B was primarily responsible for methylation of the *mTOR* gene promoter after CAB. This resulted in the activation of the PI3K/AKT/mTOR signaling pathway and overexpression of HIF-1$\alpha$ resulted in high Vascular endothelial growth factor (VEGF) expression [31]. Further research is needed to determine if *mTOR* can function as a practical therapeutic target to mitigate neovascularization during CAB therapy via the blockade of downstream pathways. Moreover, a recent study demonstrates that the histone methylation also takes part in CAB. Through lowering Forkhead-box protein O3a (FoxO3a) mediated oxidative stress, inhibition of EZH2 prevents corneal neovascularization [182]. In detail, EZH2, a core component of HMTs, is responsible for histone H3 lysine 27 trimethylation [108]. More evidence points to the possibility that methylation-inhibiting enzymes could slow the progression of several diseases.

### Corneal dystrophies

Corneal dystrophies are rare genetic disorders that impact both eyes. They occur due to the accumulation of specific substances produced in various layers of the cornea. There are different classifications of corneal dystrophies depending on anatomical structure, clinical manifestations, and inherence patterns [183, 184]. Currently, methylation studies on corneal dystrophies are mainly conducted in Fuchs endothelial cell dystrophy (FECD), with a few in Granular corneal dystrophy type 2 (GCD2) [185–187].

The most prevalent corneal endothelial dystrophy, FECD, is a significant indicator and the leading cause of corneal transplant surgeries among patients worldwide [188, 189]. FECD is a highly prevalent, progressively bilateral disease [190]. Generally, the gradual and persistent loss and dysfunction of endothelial cells in both structures and function eventually result in corneal edema [142, 191]. The corneal endothelial cells (CEnCs) are derived from the neural crest and are in a specialized extracellular mesenchyme. Many diseases that impact CEnCs can compromise corneal function and visual acuity. Therefore, it is crucial to maintain a specific physiological range of stromal hydration for clear vision [192]. FECD is linked to several spontaneous and inherited mutations, characterized by abnormal accumulation of extracellular mesenchyme, but the underlying molecular pathogenesis of it is unknown [187, 193]. DNA methylation has recently been suggested to affect corneal endothelial metabolism, cytoskeletal structure, and ion transport [186]. Some investigators have speculated that DNA methylation patterns may contribute to corneal edema and the resulting loss of corneal transparency in FECD [12]. Besides, miRNA gene promoters are often affected by abnormal DNA methylation in FECD. miRNAs, which are tiny non-coding RNAs that have undergone extensive evolutionary conservation, regulate not only fundamental biological processes including development, stress, and metabolism but also the entire course of disease development [194, 195]. The extracellular matrix (ECM) inducible genes snail and *ZEB1* are highly expressed in FECD [119]. Therefore, a study confirmed that aberrant methylation of miRNA promoters also contributes to FECD. *MiR-199B* hypermethylation completely silences the maturation transcript miR-199b-5p, increasing the expression of snai1 and ZEB1, as well as activating the transforming growth factor β (TGFβ) signaling pathway [119, 187]. These effects lead to increased ECM deposition in FECD, suggesting a methylation-regulated mechanism for ECM protein production and secretion by CEnCs.

### Other CDs

In addition to the aforementioned CDs, methylation modification has also been explored in a few other corneal conditions such as keratoconus (KTCN), brittle cornea syndrome type 2 (BCS2) and diabetic keratopathy.

KTCN is identified by progressive corneal dilation and thinning, which causes vision impairment and significantly lowers patient’s quality of life. The disease is influenced by a variety of factors, including complex gene, individual differences, and environmental effects such as ultraviolet radiation [196–198]. Nonetheless, the underlying cause or pathogenesis of KTCN remains unclear [199]. Additionally, there is growing interest in non-genetic factors, such as epigenetic factors like DNA methylation modifications, are gaining attention for their potential involvement in symptom development. To investigate the epigenetic role of KTCN, Kabza et al. performed DNA methylation sequencing and analysis of corneas affected by KTCN. The study confirmed 112 differentially methylated regions in the DNA, many of which overlapped with the sensitive sites of KTCN. Furthermore, 12 genes including *WNT5A*, *IQGAP2*, *PARVB*, *WNT3*, and *RB1* were downregulated [120, 200]. Intriguingly, a study showed that some of these genes had been downregulated in the corneas from patients with KTCN compared with non-KTCN [201]. Taken together, DNA methylation may be a possible explanation causing KTCN. However, the researchers discovered dysregulation of related genes, such as *TGFβ1*, *P4HB*, and *BCL2*, in patients with KTCN when they examined the mitochondrial DNA (mtDNA) methylation and sequencing of those genes. No differences were found in the mtDNA methylation sequencing, suggesting that the role of mtDNA methylation modifications in KTCN was not responsible for expression differences. The deeper mechanisms are worth exploring [202].

Furthermore, BCS2 is an inherited connective tissue disorder with *PRDM5* being one of the most often mutated genes [203, 204]. A study has identified H3K9me2 on the *PRDM5* target gene in CFs from BCS2 patients, suggesting that the mutation may be responsible for the activation of CFs through the histoplasmosis [205]. It appears that this mutation may exert epigenetic effects via histone methylation, resulting in irreversible ocular damage.

In addition, diabetic keratopathy is characterized by impaired CEWH, compromised barrier function, and diminished tear secretion, among other features [206]. Recent research comparing the DNA methylation patterns of limbal epithelial cells in primary cultures from diabetic and non-diabetic individuals reveals epigenetic changes in the diabetic cornea. These changes include dual inhibition of *WNT5A* through DNA methylation and miRNA activity suggesting that *WNT5A* serves as a novel stimulator for CEWH and could be a potential target for improving wound healing and stem cells in diabetic corneas [14]. Simultaneously, this provides new evidence for the significant role of methylation in CDs.

## Conclusion and prospects

### Targeting risk factor genes

The identification of aberrantly methylated CpG loci and the characterization of their distribution patterns, which are complementary diagnostic methods widely used in oncological and immunological diseases [207, 208]. Similarly, the promoters of various methylation-related key genes are closely associated with CDs, such as *mTOR* gene in CAB and miRNA gene promoters in FECD, which may be target for detection [31, 187]. This means that identifying the differentially methylated loci for some genetic CDs seems particularly crucial. Additionally, the methylation levels of specific genes in peripheral blood could serve as biomarkers for early disease diagnosis or for predicting drug efficacy. For example, this could involve conducting screenings to detect lung cancer at an early stage or evaluating the efficacy of VEGF-targeted drugs on cancer cells [209, 210]. We must acknowledge that more basic and clinical research are still needed for the application of epigenetic modifications as non-invasive biomarkers in ophthalmology diagnosis.

### Targeting metabolic synthesis

Metabolic substances such as SAM, folic acid [211] and vitamin B12 [212] are necessary for DNA and chromatin alterations, serving as methyl donors for DNA and histone methylation. According to a study conducted by Lan et al., folic acid supplementation significantly reduced the toxic effects of drugs on the cornea [213], including in a clinical trial [214]. Moreover, the methylation level is closely connected to changes in folate intake [215]. Succinate and ferredoxin can also affect DNA and histone demethylation enzymes. Specifically, high levels of succinate can inhibit DNMT activity, leading to decreased DNA methylation levels in cells [216]. Furthermore, succinate is significant in signaling pathways related to inflammation, hypoxia, and metabolism [217] that may take part in CDs. Therefore, metabolic changes may result in global changes in the methylation-associated genome. The corresponding changes in triggered metabolites may imply potential therapeutic targets, and metabolic changes can modulate specific loci and induce genes, leading to persistent epigenetic modifications that can be inherited between generations.

### Targeting enzymes

Methylation-related regulators or inhibitors can influence the expression levels of upstream and downstream factors by affecting methylation balance in their promoters. Currently, the treatment modalities for prevalent corneal pathologies primarily strive to manage inflammation, prevent infection, and improve visual acuity to the greatest extent feasible. Methylation regulatory agents have the plausible ability to supplement the extant therapies. Some researchers have hypothesized that combining anti-VEGF with DNMT inhibitors may improve the treatment of neovascularization-related ocular diseases [218, 219]. Clinically, epigenetic therapies are more targeted and cause fewer side effects than regular drugs. Some of these treatments have shown promise in treating systemic diseases by inhibiting DNMTs and may be a potential treatment for CDs. The DNMT inhibitors 5-Aza (azacytidine; Vidaza) and 5-Aza-20-deoxycytidine (decitabine; Dacron) have received FDA approval for the treatment of cutaneous T-cell lymphoma and bone marrow cancer, respectively [2, 220]. However, the use of epigenetic modalities to treat CDs is still in clinical trials. For instance, EZH2 inhibitors (EPZ-6438) are expected to become drugs for corneal scarring [110]. Future epigenetic advancements may lead to a deeper understanding of the pathophysiology of CDs. Since DNMT is associated with diseases, it is possible that DNMT modulators will help us treat CDs more effectively than current methods allow.

### Perspectives

New technological advancements have renewed interest in using methylation modifications to treat ocular diseases. Methylation, a critical biochemical process, plays a crucial role in DNA and histone modifications, corneal gene regulation and cell fate. It also affects pathways associated with CDs. Some progress has been made in studying methylation modifications in the eye, particularly in the retina. There is a growing interest among researchers in PTMs, including methylation and ubiquitination. Therefore, it is necessary to examine more closely the specific role that methylation modifications play in the mechanisms of CDs. Studies of methylation modifications on the cornea currently concentrate on corneal cell differentiation, changes in gene expression and its methylation sites in CDs, as well as changes in related regulatory factors. As a result, disease genes are enriched for important pathways and predictions. Lack of animal models for DNA methylation editing, which targets de novo DNA methylation editing by linking the action of CpGi, is the fundamental obstacle to DNA methylation investigations. To further advance our understanding of methylation modifications, in-depth studies can be conducted using single cell sequencing and triple sequencing techniques. On the one hand, it helps understand the importance of methylation modifications for eye development, which can be analyzed by methylation regulatory factors such as transferases and demethylases, as previous studies on zebrafish eye development clearly demonstrated the importance of DNMTs and dynamic expression patterns [221, 222]. On the other hand, it will enable better prognostic analyses, early screening, diagnosis, and targeted treatment of CDs. More importantly, it will provide novel treatment strategies for CDs, particularly those that are unresponsive to conventional therapies. Despite the progress made, challenges and questions remain. For example, what are the profiles of methylation regulatory factors in different types of corneal cells? How does each type of molecule change in corneal and related ocular diseases? What are the main contributing elements to complex CDs? Additionally, what roles do non-CpG methylation, m^6^A-related RNA methylation, and histone methylation play in corneal development and diseases? Is there a close correlation between the effects of methylation modifications? Finally, are there any side effects associated with the concomitant use of related inhibitors for CDs treatment? These considerations should be considered in future studies. Furthermore, it is crucial to conduct more extensive research to identify target molecules and biomarkers, elucidate the pathogenesis of epigenetically linked diseases and evaluate the safety and effectiveness of new epigenetic therapies for treating CDs.

## References

[1] Berger SL, Kouzarides T, Shiekhattar R, Shilatifard A (2009). An operational definition of epigenetics. Genes Dev.

[2] Kowluru RA, Kowluru A, Mishra M, Kumar B (2015). Oxidative stress and epigenetic modifications in the pathogenesis of diabetic retinopathy. Prog Retin Eye Res.

[3] Natarajan R (2021). Epigenetic mechanisms in diabetic vascular complications and metabolic memory: the 2020 Edwin Bierman Award Lecture. Diabetes.

[4] Li Y (2021). Modern epigenetics methods in biological research. Methods.

[5] Feinberg AP, Levchenko A (2023). Epigenetics as a mediator of plasticity in cancer. Science.

[6] Moore-Morris T, van Vliet PP, Andelfinger G, Puceat M (2018). Role of epigenetics in cardiac development and congenital diseases. Physiol Rev.

[7] Ling C, Rönn T (2019). Epigenetics in human obesity and type 2 diabetes. Cell Metab.

[8] Giles JR, Manne S, Freilich E, Oldridge DA, Baxter AE, George S (2022). Human epigenetic and transcriptional T cell differentiation atlas for identifying functional T cell-specific enhancers. Immunity.

[9] Jiang W, Zhang LX, Tan XY, Yu P, Dong M (2022). Inflammation and histone modification in chronic pain. Front Immunol.

[10] Muniyandi A, Martin M, Sishtla K, Motolani A, Sun M, Jensen NR (2023). PRMT5 is a therapeutic target in choroidal neovascularization. Sci Rep.

[11] Jung G, Hernández-Illán E, Moreira L, Balaguer F, Goel A (2020). Epigenetics of colorectal cancer: biomarker and therapeutic potential. Nat Rev Gastroenterol Hepatol.

[12] Chen E, Bohm K, Rosenblatt M, Kang K (2020). Epigenetic regulation of anterior segment diseases and potential therapeutics. Ocul Surf.

[13] Owen N, Toms M, Tian Y, Toualbi L, Richardson R, Young R (2023). Loss of the crumbs cell polarity complex disrupts epigenetic transcriptional control and cell cycle progression in the developing retina. J Pathol.

[14] Shah R, Spektor TM, Weisenberger DJ, Ding H, Patil R, Amador C (2023). Reversal of dual epigenetic repression of non-canonical Wnt-5a normalises diabetic corneal epithelial wound healing and stem cells. Diabetologia.

[15] Shahraki K, Shahraki K, Ghasemi Boroumand P, Sheervalilou R (2023). Promotor methylation in ocular surface squamous neoplasia development: epigenetics implications in molecular diagnosis. Expert Rev Mol Diagn.

[16] Ling C, Groop L (2009). Epigenetics: a molecular link between environmental factors and type 2 diabetes. Diabetes.

[17] Fiorito G, Caini S, Palli D, Bendinelli B, Saieva C, Ermini I (2021). DNA methylation-based biomarkers of aging were slowed down in a two-year diet and physical activity intervention trial: the DAMA study. Aging Cell.

[18] Loyfer N, Magenheim J, Peretz A, Cann G, Bredno J, Klochendler A (2023). A DNA methylation atlas of normal human cell types. Nature.

[19] Li T, Tan Y-T, Chen Y-X, Zheng X-J, Wang W, Liao K (2023). Methionine deficiency facilitates antitumour immunity by altering m6A methylation of immune checkpoint transcripts. Gut.

[20] Wu D, Shi Y, Zhang H, Miao C (2023). Epigenetic mechanisms of immune remodeling in sepsis: targeting histone modification. Cell Death Dis.

[21] Dai X, Ren T, Zhang Y, Nan N (2021). Methylation multiplicity and its clinical values in cancer. Expert Rev Mol Med.

[22] Menezo Y, Clement P, Clement A, Elder K (2020). Methylation: an ineluctable biochemical and physiological process essential to the transmission of life. Int J Mol Sci.

[23] Lanza M, Benincasa G, Costa D, Napoli C (2019). Clinical role of epigenetics and network analysis in eye diseases: a translational science review. J Ophthalmol.

[24] Zhang X, Zhao L, Hambly B, Bao S, Wang K (2017). Diabetic retinopathy: reversibility of epigenetic modifications and new therapeutic targets. Cell Biosci.

[25] Wang N, Yao F, Liu D, Jiang H, Xia X, Xiong S (2022). RNA N6-methyladenosine in nonocular and ocular disease. J Cell Physiol.

[26] Kumar A, Yun H, Funderburgh ML, Du Y (2022). Regenerative therapy for the Cornea. Prog Retin Eye Res.

[27] Meek KM, Knupp C (2015). Corneal structure and transparency. Prog Retin Eye Res.

[28] Hu J, Lin Y (2020). Fusarium infection alters the m6A-modified transcript landscape in the cornea. Exp Eye Res.

[29] Varanasi SK, Reddy PBJ, Bhela S, Jaggi U, Gimenez F, Rouse BT (2017). Azacytidine treatment inhibits the progression of herpes stromal keratitis by enhancing regulatory T cell function. J Virol.

[30] Shan K, Zhou R-M, Xiang J, Sun Y-N, Liu C, Lv M-W (2020). FTO regulates ocular angiogenesis via m6A-YTHDF2-dependent mechanism. Exp Eye Res.

[31] Li J, Du S, Shi Y, Han J, Niu Z, Wei L (2021). Rapamycin ameliorates corneal injury after alkali burn through methylation modification in mouse TSC1 and mTOR genes. Exp Eye Res.

[32] De Riso G, Fiorillo DFG, Fierro A, Cuomo M, Chiariotti L, Miele G (2020). Modeling DNA methylation profiles through a dynamic equilibrium between methylation and demethylation. Biomolecules.

[33] Greenberg MVC, Bourc’his D (2019). The diverse roles of DNA methylation in mammalian development and disease. Nat Rev Mol Cell Biol.

[34] De Smedt E, Lui H, Maes K, De Veirman K, Menu E, Vanderkerken K (2018). The epigenome in multiple myeloma: impact on tumor cell plasticity and drug response. Front Oncol.

[35] Li E, Zhang Y (2014). DNA methylation in mammals. Cold Spring Harb Perspect Biol.

[36] Chen T, Li E (2004). Structure and function of eukaryotic DNA methyltransferases. Curr Top Dev Biol.

[37] Moore LD, Le T, Fan G (2013). DNA methylation and its basic function. Neuropsychopharmacology.

[38] Jiang J, Yan T, Guo F (2021). Global DNA 5hmC and CK195hmC+ contents: a promising biomarker for predicting prognosis in small hepatocellular carcinoma. Curr Oncol.

[39] Madrid A, Borth LE, Hogan KJ, Hariharan N, Papale LA, Alisch RS (2021). DNA methylation and hydroxymethylation have distinct genome-wide profiles related to axonal regeneration. Epigenetics.

[40] Bird A (2002). DNA methylation patterns and epigenetic memory. Genes Dev.

[41] Jia D, Jurkowska RZ, Zhang X, Jeltsch A, Cheng X (2007). Structure of Dnmt3a bound to Dnmt3L suggests a model for de novo DNA methylation. Nature.

[42] Ito S, Shen L, Dai Q, Wu SC, Collins LB, Swenberg JA (2011). Tet proteins can convert 5-methylcytosine to 5-formylcytosine and 5-carboxylcytosine. Science.

[43] Rasmussen KD, Helin K (2016). Role of TET enzymes in DNA methylation, development, and cancer. Genes Dev.

[44] Wischnewski F, Friese O, Pantel K, Schwarzenbach H (2007). Methyl-CpG binding domain proteins and their involvement in the regulation of the MAGE-A1, MAGE-A2, MAGE-A3, and MAGE-A12 gene promoters. Mol Cancer Res.

[45] Chen Z, Zhang Y (2020). Role of mammalian DNA methyltransferases in development. Annu Rev Biochem.

[46] Smith ZD, Meissner A (2013). DNA methylation: roles in mammalian development. Nat Rev Genet.

[47] Rottach A, Frauer C, Pichler G, Bonapace IM, Spada F, Leonhardt H (2010). The multi-domain protein Np95 connects DNA methylation and histone modification. Nucleic Acids Res.

[48] Filion GJP, Zhenilo S, Salozhin S, Yamada D, Prokhortchouk E, Defossez P-A (2006). A family of human zinc finger proteins that bind methylated DNA and repress transcription. Mol Cell Biol.

[49] Guo H, Zhu P, Yan L, Li R, Hu B, Lian Y (2014). The DNA methylation landscape of human early embryos. Nature.

[50] Seisenberger S, Peat JR, Hore TA, Santos F, Dean W, Reik W (2013). Reprogramming DNA methylation in the mammalian life cycle: building and breaking epigenetic barriers. Philos Trans R Soc Lond B Biol Sci.

[51] Zhang W, Shiraishi A, Suzuki A, Zheng X, Kodama T, Ohashi Y (2004). Expression and distribution of tissue transglutaminase in normal and injured rat cornea. Curr Eye Res.

[52] Dunn BK (2003). Hypomethylation: one side of a larger picture. Ann N Y Acad Sci.

[53] Eden A, Gaudet F, Waghmare A, Jaenisch R (2003). Chromosomal instability and tumors promoted by DNA hypomethylation. Science.

[54] Pfeifer GP. Defining driver DNA methylation changes in human cancer. Int J Mol Sci. 2018;19:1166. 10.3390/ijms19041166 PMC5979276 29649096

[55] Jeong K, Murphy JM, Kim JH, Campbell PM, Park H, Rodriguez YAR (2021). FAK activation promotes SMC dedifferentiation via increased DNA methylation in contractile genes. Circ Res.

[56] Rotondo Dottore G, Lanzolla G, Comi S, Menconi F, Mencacci LC, Dallan I (2023). Insights into the role of DNA methylation and gene expression in graves orbitopathy. J Clin Endocrinol Metab.

[57] Zhao A, Li Y, Niu M, Li G, Luo N, Zhou L (2020). SNCA hypomethylation in rapid eye movement sleep behavior disorder is a potential biomarker for Parkinson’s disease. J. Parkinsons Dis.

[58] Rider CF, Carlsten C (2019). Air pollution and DNA methylation: effects of exposure in humans. Clin Epigenetics.

[59] Hughes AL, Kelley JR, Klose RJ (2020). Understanding the interplay between CpG island-associated gene promoters and H3K4 methylation. Biochim Biophys Acta Gene Regul Mech.

[60] Wang Y, Leung FCC (2004). An evaluation of new criteria for CpG islands in the human genome as gene markers. Bioinformatics.

[61] Gardiner-Garden M, Frommer M (1987). CpG islands in vertebrate genomes. J Mol Biol.

[62] Luo G, Jing X, Yang S, Peng D, Dong J, Li L (2019). DNA methylation regulates corneal epithelial wound healing by targeting miR-200a and CDKN2B. Invest Opthalmol Vis Sci.

[63] Weber M, Hellmann I, Stadler MB, Ramos L, Pääbo S, Rebhan M (2007). Distribution, silencing potential and evolutionary impact of promoter DNA methylation in the human genome. Nat Genet.

[64] Barski A, Cuddapah S, Cui K, Roh TY, Schones DE, Wang Z (2007). High-resolution profiling of histone methylations in the human genome. Cell.

[65] Zhou VW, Goren A, Bernstein BE (2011). Charting histone modifications and the functional organization of mammalian genomes. Nat Rev Genet.

[66] Jones PA (2012). Functions of DNA methylation: islands, start sites, gene bodies and beyond. Nat Rev Genet.

[67] Yang L, Ma D-W, Cao Y-P, Li D-Z, Zhou X, Feng J-F (2021). PRMT5 functionally associates with EZH2 to promote colorectal cancer progression through epigenetically repressing CDKN2B expression. Theranostics.

[68] Angeloni A, Bogdanovic O (2019). Enhancer DNA methylation: implications for gene regulation. Essays Biochem.

[69] SanMiguel JM, Bartolomei MS (2018). DNA methylation dynamics of genomic imprinting in mouse development. Biol Reprod.

[70] Héberlé É, Bardet Anaïs F (2019). Sensitivity of transcription factors to DNA methylation. Essays Biochem.

[71] Takahashi Y, Morales Valencia M, Yu Y, Ouchi Y, Takahashi K, Shokhirev MN (2023). Transgenerational inheritance of acquired epigenetic signatures at CpG islands in mice. Cell.

[72] Yang B, Wang J-Q, Tan Y, Yuan R, Chen Z-S, Zou C (2021). RNA methylation and cancer treatment. Pharmacol Res.

[73] Rana AK, Ankri S (2016). Reviving the RNA world: an insight into the appearance of RNA methyltransferases. Front Genet.

[74] Zhao BS, Roundtree IA, He C (2017). Post-transcriptional gene regulation by mRNA modifications. Nat Rev Mol Cell Biol.

[75] Wei CM, Moss B (1975). Methylated nucleotides block 5’-terminus of vaccinia virus messenger RNA. Proc Natl Acad Sci USA.

[76] Hu J, Lin Y (2020). Fusarium infection alters the m(6)A-modified transcript landscape in the cornea. Exp Eye Res.

[77] Liu P, Li F, Lin J, Fukumoto T, Nacarelli T, Hao X (2021). m(6)A-independent genome-wide METTL3 and METTL14 redistribution drives the senescence-associated secretory phenotype. Nat Cell Biol.

[78] Zhao B, Huang J, Lou X, Yao K, Ye M, Mou Q (2022). Endothelial CYP2J2 overexpression restores the BRB via METTL3-mediated ANXA1 upregulation. FASEB J.

[79] Wang P, Doxtader KA, Nam Y (2016). Structural basis for cooperative function of Mettl3 and Mettl14 methyltransferases. Mol Cell.

[80] Jia G, Fu Y, Zhao X, Dai Q, Zheng G, Yang Y (2011). N6-methyladenosine in nuclear RNA is a major substrate of the obesity-associated FTO. Nat Chem Biol.

[81] Tang S, Meng J, Tan J, Liu X, Zhou H, Li N (2022). N6-methyladenosine demethylase FTO regulates inflammatory cytokine secretion and tight junctions in retinal pigment epithelium cells. Clin Immunol.

[82] Chen XY, Zhang J, Zhu JS (2019). The role of m(6)A RNA methylation in human cancer. Mol Cancer.

[83] Qin L, Min S, Shu L, Pan H, Zhong J, Guo J (2020). Genetic analysis of N6-methyladenosine modification genes in Parkinson’s disease. Neurobiol Aging.

[84] Theler D, Dominguez C, Blatter M, Boudet J, Allain FH (2014). Solution structure of the YTH domain in complex with N6-methyladenosine RNA: a reader of methylated RNA. Nucleic Acids Res.

[85] Boo SH, Ha H, Kim YK (2022). m1A and m6A modifications function cooperatively to facilitate rapid mRNA degradation. Cell Rep.

[86] Lee Y, Choe J, Park OH, Kim YK (2020). Molecular mechanisms driving mRNA degradation by m6A modification. Trends Genet.

[87] Biggar KK, Li SS-C (2015). Non-histone protein methylation as a regulator of cellular signalling and function. Nat Rev Mol Cell Biol.

[88] Lanouette S, Mongeon V, Figeys D, Couture J-F (2014). The functional diversity of protein lysine methylation. Mol Syst Biol.

[89] Wu Q, Li J, Sun S, Chen X, Zhang H, Li B (2017). YAP/TAZ-mediated activation of serine metabolism and methylation regulation is critical for LKB1-deficient breast cancer progression. Biosci Rep.

[90] Talbert PB, Henikoff S (2021). Histone variants at a glance. J Cell Sci.

[91] Bentley GA, Lewit-Bentley A, Finch JT, Podjarny AD, Roth M (1984). Crystal structure of the nucleosome core particle at 16 A resolution. J Mol Biol.

[92] Luger K, Rechsteiner TJ, Flaus AJ, Waye MM, Richmond TJ (1997). Characterization of nucleosome core particles containing histone proteins made in bacteria. J Mol Biol.

[93] Daskalaki MG, Tsatsanis C, Kampranis SC (2018). Histone methylation and acetylation in macrophages as a mechanism for regulation of inflammatory responses. J Cell Physiol.

[94] Martin C, Zhang Y (2005). The diverse functions of histone lysine methylation. Nat Rev Mol Cell Biol.

[95] Smith BC, Denu JM (2009). Chemical mechanisms of histone lysine and arginine modifications. Biochim Biophys Acta.

[96] Jørgensen S, Eskildsen M, Fugger K, Hansen L, Larsen MSY, Kousholt AN (2011). SET8 is degraded via PCNA-coupled CRL4(CDT2) ubiquitylation in S phase and after UV irradiation. J Cell Biol.

[97] Yang M, Gocke CB, Luo X, Borek D, Tomchick DR, Machius M (2006). Structural basis for CoREST-dependent demethylation of nucleosomes by the human LSD1 histone demethylase. Mol Cell.

[98] Tsukada Y, Fang J, Erdjument-Bromage H, Warren ME, Borchers CH, Tempst P (2006). Histone demethylation by a family of JmjC domain-containing proteins. Nature.

[99] Shi Y, Lan F, Matson C, Mulligan P, Whetstine JR, Cole PA (2004). Histone demethylation mediated by the nuclear amine oxidase homolog LSD1. Cell.

[100] Jambhekar A, Dhall A, Shi Y (2019). Roles and regulation of histone methylation in animal development. Nat Rev Mol Cell Biol.

[101] Zhang Y, Chen J, Liu H, Mi R, Huang R, Li X (2022). The role of histone methylase and demethylase in antitumor immunity: a new direction for immunotherapy. Front Immunol.

[102] Rao RC, Tchedre KT, Malik MTA, Coleman N, Fang Y, Marquez VE (2010). Dynamic patterns of histone lysine methylation in the developing retina. Invest Ophthalmol Vis Sci.

[103] Levy D, Kuo AJ, Chang Y, Schaefer U, Kitson C, Cheung P (2011). Lysine methylation of the NF-κB subunit RelA by SETD6 couples activity of the histone methyltransferase GLP at chromatin to tonic repression of NF-κB signaling. Nat Immunol.

[104] Shi X, Kachirskaia I, Yamaguchi H, West LE, Wen H, Wang EW (2007). Modulation of p53 function by SET8-mediated methylation at lysine 382. Mol Cell.

[105] Rao RC, Chen DF, Miller JW (2011). An epigenetic approach toward understanding ocular α-herpesvirus pathogenesis and treatment. Int Ophthalmol Clin.

[106] Horswill MA, Narayan M, Warejcka DJ, Cirillo LA, Twining SS (2008). Epigenetic silencing of maspin expression occurs early in the conversion of keratocytes to fibroblasts. Exp Eye Res.

[107] Wan S, Zhou Y, Huang Q, Yang Y (2021). Dot1l aggravates keratitis induced by herpes simplex virus type 1 in mice via p38 MAPK-mediated oxidative stress. Oxid Med Cell Longev.

[108] Zhu Y-N, Gan X-W, Pan F, Ni X-T, Myatt L, Wang W-S (2022). Role of EZH2-mediated H3K27me3 in placental ADAM12-S expression: implications for fetoplacental growth. BMC Med.

[109] Chen Q, Zheng P-S, Yang W-T (2016). EZH2-mediated repression of GSK-3β and TP53 promotes Wnt/β-catenin signaling-dependent cell expansion in cervical carcinoma. Oncotarget.

[110] Liao K, Cui Z, Zeng Y, Liu J, Wang Y, Wang Z (2021). Inhibition of enhancer of zeste homolog 2 prevents corneal myofibroblast transformation in vitro. Exp Eye Res.

[111] Koch A, Joosten SC, Feng Z, de Ruijter TC, Draht MX, Melotte V (2018). Analysis of DNA methylation in cancer: location revisited. Nat Rev Clin Oncol.

[112] Siu MT, Weksberg R (2017). Epigenetics of autism spectrum disorder. Adv Exp Med Biol.

[113] Ni Y, Zhang H, Chu L, Zhao Y (2023). m6A modification-association with oxidative stress and implications on eye diseases. Antioxidants.

[114] Engel M, Eggert C, Kaplick PM, Eder M, Röh S, Tietze L (2018). The role of m6A/m-RNA methylation in stress response regulation. Neuron.

[115] Tognini P, Napoli D, Tola J, Silingardi D, Della Ragione F, D’Esposito M (2015). Experience-dependent DNA methylation regulates plasticity in the developing visual cortex. Nat Neurosci.

[116] Ouyang J, Sun W, Shen H, Liu X, Wu Y, Jiang H (2023). Truncation mutations in MYRF underlie primary angle closure glaucoma. Hum Genet.

[117] Meng J, Liu X, Tang S, Liu Y, Zhao C, Zhou Q (2022). METTL3 inhibits inflammation of retinal pigment epithelium cells by regulating NR2F1 in an m6A-dependent manner. Front Immunol.

[118] Bonnin N, Belville C, Chiambaretta F, Sapin V, Blanchon L (2014). DNA methyl transferases are differentially expressed in the human anterior eye segment. Acta Ophthalmol.

[119] Okumura N, Minamiyama R, Ho LT, Kay EP, Kawasaki S, Tourtas T (2015). Involvement of ZEB1 and Snail1 in excessive production of extracellular matrix in Fuchs endothelial corneal dystrophy. Lab Invest.

[120] Kabza M, Karolak JA, Rydzanicz M, Udziela M, Gasperowicz P, Ploski R (2019). Multiple differentially methylated regions specific to keratoconus explain known keratoconus linkage loci. Invest Opthalmol Vis Sci.

[121] Mohan RR, Martin LM, Sinha NR (2021). Novel insights into gene therapy in the cornea. Exp Eye Res.

[122] Ma J, Wang Y, Wei P, Jhanji V (2018). Biomechanics and structure of the cornea: implications and association with corneal disorders. Surv Ophthalmol.

[123] Almubrad T, Akhtar S (2011). Structure of corneal layers, collagen fibrils, and proteoglycans of tree shrew cornea. Mol Vis.

[124] Masterton S, Ahearne M (2018). Mechanobiology of the corneal epithelium. Exp Eye Res.

[125] Sasamoto Y, Wu S, Lee CAA, Jiang JY, Ksander BR, Frank MH (2023). Epigenetic regulation of corneal epithelial differentiation by TET2. Int J Mol Sci.

[126] Hayashi R, Ishikawa Y, Ito M, Kageyama T, Takashiba K, Fujioka T (2012). Generation of corneal epithelial cells from induced pluripotent stem cells derived from human dermal fibroblast and corneal limbal epithelium. PLoS ONE.

[127] Sareen D, Saghizadeh M, Ornelas L, Winkler MA, Narwani K, Sahabian A (2014). Differentiation of human limbal-derived induced pluripotent stem cells into limbal-like epithelium. Stem Cells Transl Med.

[128] Nishikiori N, Sawada N, Ohguro H (2008). Prevention of murine experimental corneal trauma by epigenetic events regulating claudin 6 and claudin 9. Jpn J Ophthalmol.

[129] Verma S, Singh A, Varshney A, Chandru RA, Acharya M, Rajput J (2021). Infectious keratitis: an update on role of epigenetics. Front Immunol.

[130] Conrady CD, Jones H, Zheng M, Carr DJJ (2011). A functional type I interferon pathway drives resistance to cornea herpes simplex virus type 1 infection by recruitment of leukocytes. J Biomed Res.

[131] Riau AK, Wong TT, Lan W, Finger SN, Chaurasia SS, Hou AH (2011). Aberrant DNA methylation of matrix remodeling and cell adhesion related genes in pterygium. PLoS ONE.

[132] Winkler R, Gillis E, Lasman L, Safra M, Geula S, Soyris C (2019). m6A modification controls the innate immune response to infection by targeting type I interferons. Nat Immunol.

[133] Zhao X, Guan J-L (2011). Focal adhesion kinase and its signaling pathways in cell migration and angiogenesis. Adv Drug Deliv Rev.

[134] Yao MD, Jiang Q, Ma Y, Liu C, Zhu CY, Sun YN (2020). Role of METTL3-dependent N(6)-methyladenosine mRNA modification in the promotion of angiogenesis. Mol Ther.

[135] Lu L, Reinach PS, Kao WW (2001). Corneal epithelial wound healing. Exp Biol Med.

[136] Dai Y, Cheng M, Zhang S, Ling R, Wen J, Cheng Y (2021). METTL3-mediated m6A RNA modification regulates corneal injury repair. Stem Cells Int.

[137] Gonzalez G, Sasamoto Y, Ksander BR, Frank MH, Frank NY. Limbal stem cells: identity, developmental origin, and therapeutic potential. Wiley Interdiscip Rev Dev Biol. 2018;7:e303. 10.1002/wdev.303 PMC5814333 29105366

[138] Maltseva O, Folger P, Zekaria D, Petridou S, Masur SK (2001). Fibroblast growth factor reversal of the corneal myofibroblast phenotype. Invest Ophthalmol Vis Sci.

[139] Jester JV, Barry-Lane PA, Cavanagh HD, Petroll WM (1996). Induction of alpha-smooth muscle actin expression and myofibroblast transformation in cultured corneal keratocytes. Cornea..

[140] Ngamkitidechakul C, Burke JM, O’Brien WJ, Twining SS (2001). Maspin: synthesis by human cornea and regulation of in vitro stromal cell adhesion to extracellular matrix. Invest Ophthalmol Vis Sci.

[141] Futscher BW, Oshiro MM, Wozniak RJ, Holtan N, Hanigan CL, Duan H (2002). Role for DNA methylation in the control of cell type specific maspin expression. Nat Genet.

[142] Tan DTH, Dart JKG, Holland EJ, Kinoshita S (2012). Corneal transplantation. Lancet.

[143] Du Z, Liu M, Wang Z, Lin Z, Feng Y, Tian D (2021). EZH2-mediated inhibition of KLF14 expression promotes HSCs activation and liver fibrosis by downregulating PPARγ. Cell Prolif.

[144] Kerr K, McAneney H, McKnight AJ (2019). Differential methylation in rare ophthalmic disorders: a systematic review protocol. Syst Rev.

[145] Martin EM, Fry RC (2018). Environmental influences on the epigenome: exposure- associated DNA methylation in human populations. Annu Rev Public Health.

[146] Ferrari L, Monti P, Favero C, Carugno M, Tarantini L, Maggioni C (2022). Association between night shift work and methylation of a subset of immune-related genes. Front Public Health.

[147] Fernández-Carrión R, Sorlí JV, Asensio EM, Pascual EC, Portolés O, Alvarez-Sala A, et al. DNA-methylation signatures of tobacco smoking in a high cardiovascular risk population: modulation by the Mediterranean diet. Int J Environ Res Public Health. 2023;20:3635. 10.3390/ijerph20043635 PMC9964856 36834337

[148] Baker-Andresen D, Ratnu VS, Bredy TW (2013). Dynamic DNA methylation: a prime candidate for genomic metaplasticity and behavioral adaptation. Trends Neurosci.

[149] Meyer KD, Jaffrey SR (2014). The dynamic epitranscriptome: N6-methyladenosine and gene expression control. Nat Rev Mol Cell Biol.

[150] Ito S, D’Alessio AC, Taranova OV, Hong K, Sowers LC, Zhang Y (2010). Role of Tet proteins in 5mC to 5hmC conversion, ES-cell self-renewal and inner cell mass specification. Nature.

[151] Li D, Guo B, Wu H, Tan L, Lu Q (2015). TET family of dioxygenases: crucial roles and underlying mechanisms. Cytogenet Genome Res.

[152] Ma C, Seong H, Liu Y, Yu X, Xu S, Li Y (2021). Ten-eleven translocation proteins (TETs): tumor suppressors or tumor enhancers?. Front Biosci.

[153] He L, Huang H, Bradai M, Zhao C, You Y, Ma J (2022). DNA methylation-free Arabidopsis reveals crucial roles of DNA methylation in regulating gene expression and development. Nat Commun.

[154] Bostick M, Kim JK, Estève PO, Clark A, Pradhan S, Jacobsen SE (2007). UHRF1 plays a role in maintaining DNA methylation in mammalian cells. Science.

[155] Li X, Ma B, Liao M, Li L, Zhang X, Du M (2022). Potential impact of N6-methyladenosine RNA methylation on vision function and the pathological processes of ocular diseases: new discoveries and future perspectives. Front Biosci.

[156] Li H-B, Tong J, Zhu S, Batista PJ, Duffy EE, Zhao J (2017). m6A mRNA methylation controls T cell homeostasis by targeting the IL-7/STAT5/SOCS pathways. Nature.

[157] Wang Y, Grenell A, Zhong F, Yam M, Hauer A, Gregor E (2018). Metabolic signature of the aging eye in mice. Neurobiol Aging.

[158] He J, Neumann D, Kakazu A, Pham TL, Musarrat F, Cortina MS (2017). PEDF plus DHA modulate inflammation and stimulate nerve regeneration after HSV-1 infection. Exp Eye Res.

[159] Rowe AM, St. Leger AJ, Jeon S, Dhaliwal DK, Knickelbein JE, Hendricks RL (2013). Herpes keratitis. Prog Retin Eye Res.

[160] Rechenchoski DZ, Faccin-Galhardi LC, Linhares REC, Nozawa C (2017). Herpesvirus: an underestimated virus. Folia Microbiol.

[161] Yu T, Schuette F, Christofi M, Forrester JV, Graham GJ, Kuffova L (2022). The atypical chemokine receptor-2 fine-tunes the immune response in herpes stromal keratitis. Front Immunol.

[162] Bloom DC, Giordani NV, Kwiatkowski DL (2010). Epigenetic regulation of latent HSV-1 gene expression. Biochim Biophys Acta.

[163] Niemialtowski MG, Rouse BT (1992). Predominance of Th1 cells in ocular tissues during herpetic stromal keratitis. J Immunol.

[164] Hendricks RL, Tumpey TM, Finnegan A (1992). IFN-gamma and IL-2 are protective in the skin but pathologic in the corneas of HSV-1-infected mice. J Immunol.

[165] Suryawanshi A, Veiga-Parga T, Rajasagi NK, Reddy PBJ, Sehrawat S, Sharma S (2011). Role of IL-17 and Th17 cells in herpes simplex virus-induced corneal immunopathology. J Immunol.

[166] Sehrawat S, Suvas S, Sarangi PP, Suryawanshi A, Rouse BT (2008). In vitro-generated antigen-specific CD4+ CD25+ Foxp3+ regulatory T cells control the severity of herpes simplex virus-induced ocular immunoinflammatory lesions. J Virol.

[167] Polansky JK, Schreiber L, Thelemann C, Ludwig L, Krüger M, Baumgrass R (2010). Methylation matters: binding of Ets-1 to the demethylated Foxp3 gene contributes to the stabilization of Foxp3 expression in regulatory T cells. J Mol Med.

[168] Kim HP, Leonard WJ (2007). CREB/ATF-dependent T cell receptor-induced FoxP3 gene expression: a role for DNA methylation. J Exp Med.

[169] Uchino Y, Kawakita T, Miyazawa M, Ishii T, Onouchi H, Yasuda K (2012). Oxidative stress induced inflammation initiates functional decline of tear production. PLoS ONE.

[170] Qian Y, Wu J (1998). [The role of oxygen free radical in experimental keratitis]. [Zhonghua Yan Ke Za Zhi] Chin J Ophthalmol.

[171] Farooq Z, Banday S, Pandita TK, Altaf M (2016). The many faces of histone H3K79 methylation. Mutat Res Rev Mutat Res.

[172] Nguyen AT, Zhang Y (2011). The diverse functions of Dot1 and H3K79 methylation. Genes Dev.

[173] Zieske JD (2000). Expression of cyclin-dependent kinase inhibitors during corneal wound repair. Prog Retin Eye Res.

[174] Park J-H, Kim J-Y, Kim DJ, Kim M, Chang M, Chuck RS (2017). Effect of nitric oxide on human corneal epithelial cell viability and corneal wound healing. Sci Rep.

[175] Yang S, Chen W, Jin S, Luo G, Jing X, Liu Q (2022). SUV39H1 regulates corneal epithelial wound healing via H3K9me3-mediated repression of p27. Eye Vis.

[176] Luo G, Xu W, Chen X, Xu W, Yang S, Wang J (2023). The RNA m5C methylase NSUN2 modulates corneal epithelial wound healing. Invest Ophthalmol Vis Sci.

[177] Bizrah M, Yusuf A, Ahmad S (2019). An update on chemical eye burns. Eye.

[178] Ozge G, Karaca U, Savran M, Usta G, Gulle K, Sevimli M (2023). Salubrinal ameliorates inflammation and neovascularization via the caspase 3/Enos signaling in an alkaline-induced rat corneal neovascularization model. Medicina.

[179] Wagoner MD (1997). Chemical injuries of the eye: current concepts in pathophysiology and therapy. Surv Ophthalmol.

[180] Kwon YS, Hong HS, Kim JC, Shin JS, Son Y (2005). Inhibitory effect of rapamycin on corneal neovascularization in vitro and in vivo. Invest Ophthalmol Vis Sci.

[181] Shin YJ, Hyon JY, Choi WS, Yi K, Chung E-S, Chung T-Y (2013). Chemical injury-induced corneal opacity and neovascularization reduced by rapamycin via TGF-β1/ERK pathways regulation. Invest Ophthalmol Vis Sci.

[182] Wan S-S, Pan Y-M, Yang W-J, Rao Z-Q, Yang Y-N (2020). Inhibition of EZH2 alleviates angiogenesis in a model of corneal neovascularization by blocking FoxO3a-mediated oxidative stress. FASEB J.

[183] Weiss JS (2009). Corneal dystrophy classification. Ophthalmology.

[184] Vincent AL (2014). Corneal dystrophies and genetics in the International Committee for Classification of Corneal Dystrophies era: a review. Clin Exp Ophthalmol.

[185] Maeng Y-S, Lee G-H, Choi S-I, Kim KS, Kim EK (2015). Histone methylation levels correlate with TGFBIp and extracellular matrix gene expression in normal and granular corneal dystrophy type 2 corneal fibroblasts. BMC Med Genomics.

[186] Khuc E, Bainer R, Wolf M, Clay SM, Weisenberger DJ, Kemmer J (2017). Comprehensive characterization of DNA methylation changes in Fuchs endothelial corneal dystrophy. PLoS ONE.

[187] Pan P, Weisenberger DJ, Zheng S, Wolf M, Hwang DG, Rose-Nussbaumer JR (2019). Aberrant DNA methylation of miRNAs in Fuchs endothelial corneal dystrophy. Sci Rep.

[188] Matthaei M, Hribek A, Clahsen T, Bachmann B, Cursiefen C, Jun AS (2019). Fuchs endothelial corneal dystrophy: clinical, genetic, pathophysiologic, and therapeutic aspects. Annu Rev Vis Sci.

[189] Musch DC, Niziol LM, Stein JD, Kamyar RM, Sugar A (2011). Prevalence of corneal dystrophies in the United States: estimates from claims data. Invest Ophthalmol Vis Sci.

[190] Aiello F, Gallo Afflitto G, Ceccarelli F, Cesareo M, Nucci C (2022). Global prevalence of Fuchs endothelial corneal dystrophy (FECD) in adult population: a systematic review and meta-analysis. J Ophthalmol.

[191] Adamis AP, Filatov V, Tripathi BJ, Tripathi RC (1993). Fuchs’ endothelial dystrophy of the cornea. Surv Ophthalmol.

[192] Jeang LJ, Margo CE, Espana EM (2021). Diseases of the corneal endothelium. Exp Eye Res.

[193] Magovern M, Beauchamp GR, McTigue JW, Fine BS, Baumiller RC (1979). Inheritance of Fuchs’ combined dystrophy. Ophthalmology.

[194] Sengupta D, Deb M, Kar S, Pradhan N, Parbin S, Kirtana R (2021). Dissecting miRNA facilitated physiology and function in human breast cancer for therapeutic intervention. Semin Cancer Biol.

[195] Vidigal JA, Ventura A (2015). The biological functions of miRNAs: lessons from in vivo studies. Trends Cell Biol.

[196] McMonnies CW (2014). Epigenetic mechanisms might help explain environmental contributions to the pathogenesis of keratoconus. Eye Contact Lens.

[197] Meyer JJ, Gokul A, Vellara HR, McGhee CNJ (2023). Progression of keratoconus in children and adolescents. Br J Ophthalmol.

[198] Bykhovskaya Y, Rabinowitz YS (2021). Update on the genetics of keratoconus. Exp Eye Res.

[199] Lucas SEM, Burdon KP (2020). Genetic and environmental risk factors for keratoconus. Annu Rev Vis Sci.

[200] Meissner A, Gnirke A, Bell GW, Ramsahoye B, Lander ES, Jaenisch R (2005). Reduced representation bisulfite sequencing for comparative high-resolution DNA methylation analysis. Nucleic Acids Res.

[201] Kabza M, Karolak JA, Rydzanicz M, Szcześniak MW, Nowak DM, Ginter-Matuszewska B (2017). Collagen synthesis disruption and downregulation of core elements of TGF-β, Hippo, and Wnt pathways in keratoconus corneas. Eur J Hum Genet.

[202] Nowak-Malczewska DM, Karolak JA, Swierkowska J, Jaworska MM, Kulinska KI, Polakowski P (2021). Changes in nuclear gene expression related to mitochondrial function affect extracellular matrix, collagens, and focal adhesion in keratoconus. Transl Vis Sci Technol.

[203] Burkitt Wright EMM, Porter LF, Spencer HL, Clayton-Smith J, Au L, Munier FL (2013). Brittle cornea syndrome: recognition, molecular diagnosis and management. Orphanet J Rare Dis.

[204] Burkitt Wright EMM, Spencer HL, Daly SB, Manson FDC, Zeef LAH, Urquhart J (2011). Mutations in PRDM5 in brittle cornea syndrome identify a pathway regulating extracellular matrix development and maintenance. Am J Hum Genet.

[205] Porter LF, Galli GG, Williamson S, Selley J, Knight D, Elcioglu N (2015). A role for repressive complexes and H3K9 di-methylation in PRDM5-associated brittle cornea syndrome. Hum Mol Genet.

[206] Shah R, Amador C, Tormanen K, Ghiam S, Saghizadeh M, Arumugaswami V (2021). Systemic diseases and the cornea. Exp Eye Res.

[207] Zhao M, Zhou Y, Zhu B, Wan M, Jiang T, Tan Q (2016). IFI44L promoter methylation as a blood biomarker for systemic lupus erythematosus. Ann Rheum Dis.

[208] Xi Y, Lin Y, Guo W, Wang X, Zhao H, Miao C (2022). Multi-omic characterization of genome-wide abnormal DNA methylation reveals diagnostic and prognostic markers for esophageal squamous-cell carcinoma. Signal Transduct Target Ther.

[209] Kim J, Hwang J, Jeong H, Song H-J, Shin J, Hur G (2012). Promoter methylation status of VEGF receptor genes: a possible epigenetic biomarker to anticipate the efficacy of intracellular-acting VEGF-targeted drugs in cancer cells. Epigenetics.

[210] Hulbert A, Jusue-Torres I, Stark A, Chen C, Rodgers K, Lee B (2017). Early detection of lung cancer using DNA promoter hypermethylation in plasma and sputum. Clin Cancer Res.

[211] Ondičová M, Irwin RE, Thursby S-J, Hilman L, Caffrey A, Cassidy T (2022). Folic acid intervention during pregnancy alters DNA methylation, affecting neural target genes through two distinct mechanisms. Clin Epigenetics.

[212] An Y, Feng L, Zhang X, Wang Y, Wang Y, Tao L (2019). Dietary intakes and biomarker patterns of folate, vitamin B6, and vitamin B12 can be associated with cognitive impairment by hypermethylation of redox-related genes NUDT15 and TXNRD1. Clin Epigenetics.

[213] Gorovoy I, Prechanond T, Abia M, Afshar AR, Stewart JM (2013). Toxic corneal epitheliopathy after intravitreal methotrexate and its treatment with oral folic acid. Cornea.

[214] Hasan N, Narde HK, Das AK, Chawla R (2022). Unusual presentation of cornea verticillata with intravitreal methotrexate in a case of primary intraocular lymphoma. BMJ Case Rep.

[215] Crider KS, Yang TP, Berry RJ, Bailey LB (2012). Folate and DNA methylation: a review of molecular mechanisms and the evidence for folate’s role. Adv Nutr.

[216] Cui W, Huang Z, Pfeifer GP (2022). Lack of major genome-wide DNA methylation changes in succinate-treated human epithelial cells. Int J Mol Sci.

[217] Murphy MP, O’Neill LAJ (2018). Krebs cycle reimagined: the emerging roles of succinate and itaconate as signal transducers. Cell.

[218] Cai C, Meng C, He S, Gu C, Lhamo T, Draga D (2022). DNA methylation in diabetic retinopathy: pathogenetic role and potential therapeutic targets. Cell Biosci.

[219] Young BK, Hwang M, Johnson MW, Besirli CG, Wubben TJ (2022). A caveat about financial incentives for anti-vascular endothelial growth factor therapy for diabetic retinopathy. Am J Ophthalmol.

[220] Song SH, Han SW, Bang YJ (2011). Epigenetic-based therapies in cancer: progress to date. Drugs.

[221] Tittle RK, Sze R, Ng A, Nuckels RJ, Swartz ME, Anderson RM (2011). Uhrf1 and Dnmt1 are required for development and maintenance of the zebrafish lens. Dev Biol.

[222] Rai K, Jafri IF, Chidester S, James SR, Karpf AR, Cairns BR (2010). Dnmt3 and G9a cooperate for tissue-specific development in zebrafish. J Biol Chem.

